# Anomalous Presentation of Venous Malformations in an Adolescent Male

**DOI:** 10.7759/cureus.15756

**Published:** 2021-06-19

**Authors:** Cees T Whisonant, Shawhin R Shahriari, Joshua L Harrison, Ashley E Ederle, Gregory L Borah, Anil K Shetty

**Affiliations:** 1 Division of Plastics, Hand, Reconstructive & Burn Surgery, Department of Surgery, University of New Mexico School of Medicine, Albuquerque, USA; 2 Internal Medicine, University of New Mexico School of Medicine, Albuquerque, USA

**Keywords:** vascular anomalies, venous malformations, plastic and reconstructive surgery, glomuvenous malformations, plastic surgery

## Abstract

Venous malformations (VMs) may manifest clinically in a broad spectrum. Most VMs are sporadic with previous studies reporting less than 1.2% to be inherited. Conversely, multifocal lesions, such as glomuvenous malformations (GVMs), which have glomus cells in their vascular walls, have been reported to have a frequency of inheritance of 63.8%. Both VMs and GVMs may occur due to sporadic mutation and must be differentiated clinically because this will dictate their proper treatment. Sporadic GVMs involve skin and subcutis, with bluish-purple coloration, are painful to compression, and have no radiographic evidence of phleboliths. Previous studies have demonstrated that VMs are almost always associated with a single lesion that is nontender to compression and are often able to be diagnosed by the presence of phleboliths on radiographic imaging. We present a case of a 14-year-old right-hand-dominant male who presented with two distinct VMs on the dorsum of the right index finger at the proximal and middle phalanges. A previously biopsied lesion overlying the ipsilateral olecranon, which was reported as a possible glomus tumor versus vascular malformation, was present as well. Based on history, physical examination, multicentric presentation, and radiographic findings, the presumptive diagnosis was that the lesions were GVMs. However, after surgical excision and histopathologic examination, the lesions were determined to be VMs because of the absence of glomus cells. Due to the difference in treatment modalities for VMs and GVMs, the ability to accurately diagnose these lesions clinically is essential. This case represents an anomalous presentation of multiple venous malformations occurring in two distinct locations in a 14-year-old boy.

## Introduction

Vascular anomalies are often classified into two distinct categories, tumors versus malformations, based upon clinical and histologic characteristics. While proliferating endothelium is suggestive of a vascular tumor, malformations are described as structural anomalies [1]. A variety of distinct vascular malformations exist, which include venous malformations (VMs) and glomuvenous malformations (GVMs).

VMs are present at birth and described as blue, soft, compressible lesions that may contain palpable calcified phleboliths [2]. Errors in vascular morphogenesis lead to lesions composed of thin-walled dilated veins with abnormal smooth muscle [3]. The majority of lesions involve the skin, mucosa, and subcutaneous tissue; however, approximately half of these lesions affect deeper structures as well. VMs are typically sporadic and solitary in over 90% of patients and more than 99% are usually larger than 5 cm [4].

In contrast, GVMs are due to a loss of function mutation in the glomulin gene. GVMs are characterized by abnormal glomus cells along ectatic veins most commonly manifesting as multiple, small (<5 cm) lesions involving the skin and subcutaneous tissue [5,6]. While VMs are almost always associated with a single lesion and are often able to be diagnosed by the presence of phleboliths on radiographic imaging, GVMs present as multiple, sporadic, bluish, ovaloid nodules that are painful with compression and no evidence of phleboliths on imaging [2,4,7].

Differentiating between GVMs and VMs is of particular importance when it comes to treatment strategies and response. VMs are often difficult to excise completely due to the invasion of surrounding tissues and deeper structures, but they may be improved by external compression and are often shrinkable with sclerotherapy. There is no role for sclerotherapy or compression garments in GVMs; however, they are often easily resectable due to their more superficial nature [4].

This report demonstrates an anomalous case of VMs in an adolescent boy presenting with multiple, sporadic VMs that were unable to be differentiated clinically.

This article was previously presented as a virtual poster presentation at the Mountain West Society of Plastic Surgery Annual Meeting on March 6, 2021.

## Case presentation

**Figure 1:**
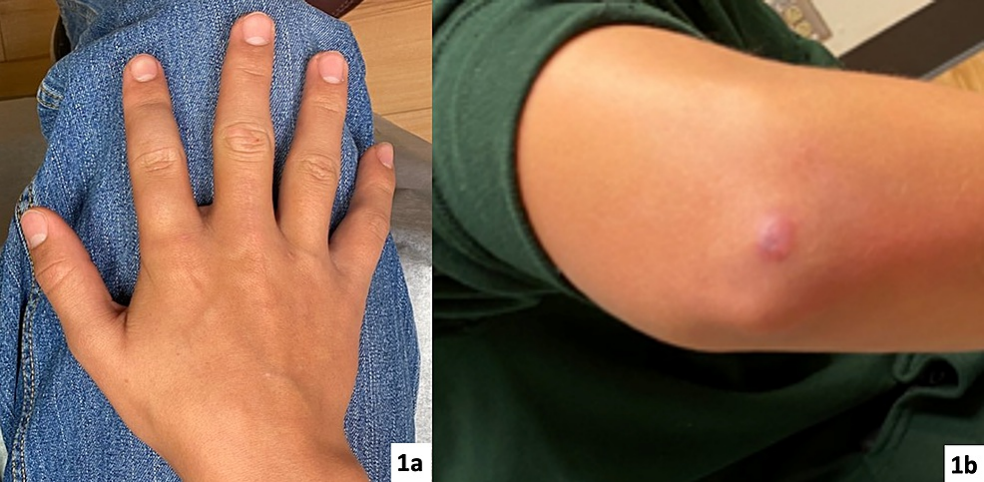
Clinical presentation (1a) Right index finger with two nodules on the dorsum of the proximal and middle phalanges. (1b) Right elbow with the previously biopsied vascular lesion; glomus tumor versus vascular malformation differentiation could not be established.

An otherwise healthy, 14-year-old, right-hand-dominant male presented to our clinic for the evaluation and treatment of two distinct, blue, subcutaneous, non-tender, mobile, ovaloid nodules overlying the dorsal aspect of his right index finger (Figure 1a).

**Figure 2:**
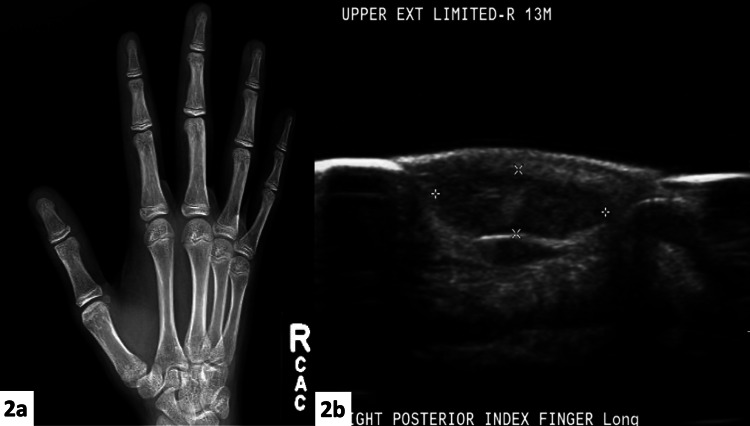
Radiographic images (2a) Plain film anteroposterior view of the right hand demonstrating no osseous changes. (2b) Ultrasound of the right index finger demonstrating a slow-flow lesion possibly representing a glomus tumor.

He endorsed a history of multiple lesions being present in different locations since birth that had been followed up clinically into adolescence. Several of these lesions were noted to regress on their own, but others grew proportionally with the patient. The lesions that exhibited proportional growth with the patient included the two on his right index finger, as well as one overlying the ipsilateral olecranon (Figure 1b). The lesion overlying the olecranon had been biopsied previously, and the histopathology demonstrated vessel walls that stained positive for the smooth muscle actin (SMA) stain and were negative for CD31. This suggested a possible GVM or VM. He had no family history of birthmarks or vascular malformations. Plain film radiographs and sonograms were obtained to further investigate the dorsal index finger lesions, which were concerning for a possible GVM. Plain films indicated no underlying osseous changes or soft tissue calcifications (i.e., no phleboliths) (Figure 2a). Ultrasound demonstrated soft tissue lesions with doppler showing no significant flow interpreted to represent a possible glomus tumor (Figure 2b).

**Figure 3:**
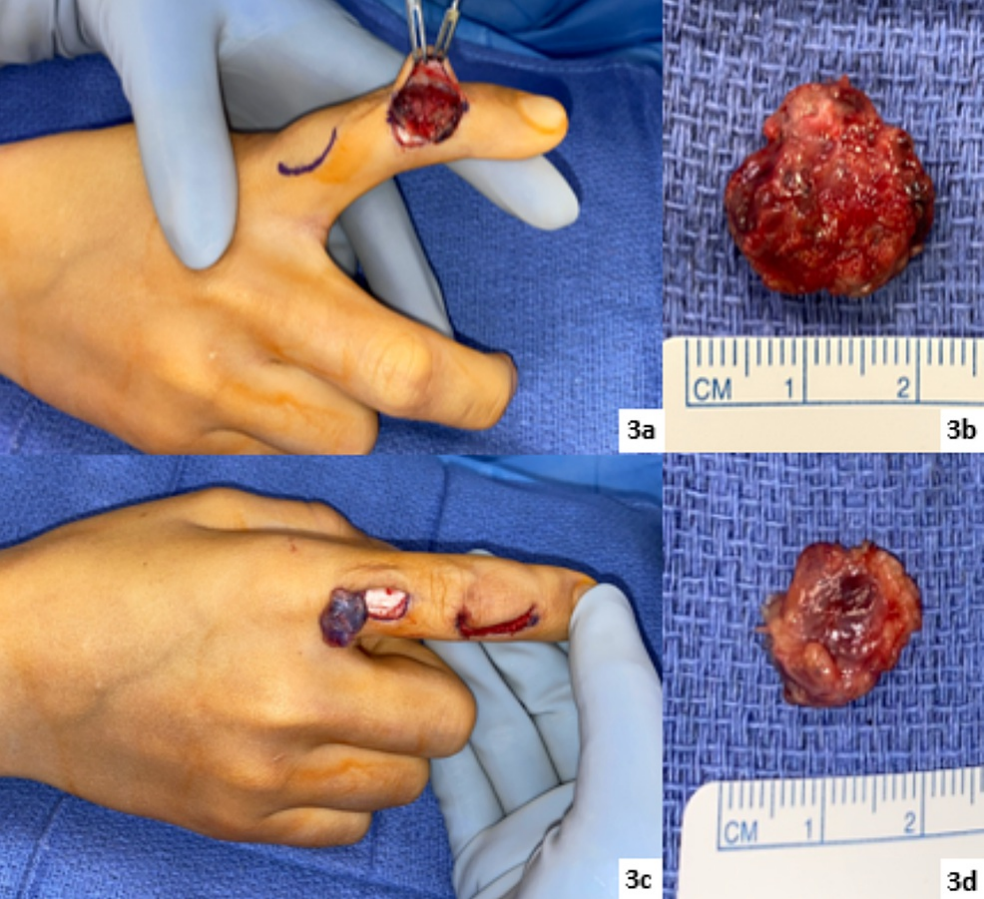
Intraoperative images (3a) Right index finger middle phalanx lesion. (3b) Right index finger middle phalanx specimen measuring 1.4 x 1.2 x 0.4 cm. (3c) Right index finger proximal phalanx lesion. (3d) Right index finger proximal phalanx specimen measuring 1.8 x 1.8 x 0.6 cm.

Based upon the superficial nature of the lesions, as well as clinical suspicion for a GVM, the patient was not considered a candidate for sclerotherapy. He underwent successful debulking of the right index finger lesions (Figure 3), and surgical specimens were sent for further histopathologic analysis.

**Figure 4:**
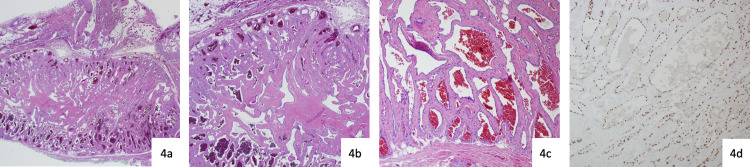
Histopathologic analysis (4a) A low-power image of the central nodule within the subcutis surrounded by uninvolved fibroadipose tissue. (4b) A nodule composed of thin-walled vessels containing erythrocytes within a sclerotic stroma. (4c) A higher power image showing the same. A few smooth muscle bundles are identified but no glomus cells are present. (4d) Immunostain for ERG is positive within the endothelial nuclei.

Histopathology demonstrated nodules composed of thin-walled vessels containing erythrocytes within the subcutis surrounded by uninvolved fibroadipose tissue (Figure 4). A few smooth muscle bundles were identified, but no glomus cells were present. SMA immunostaining for glomus cells was done with no definitive glomus cells seen.

**Figure 5:**
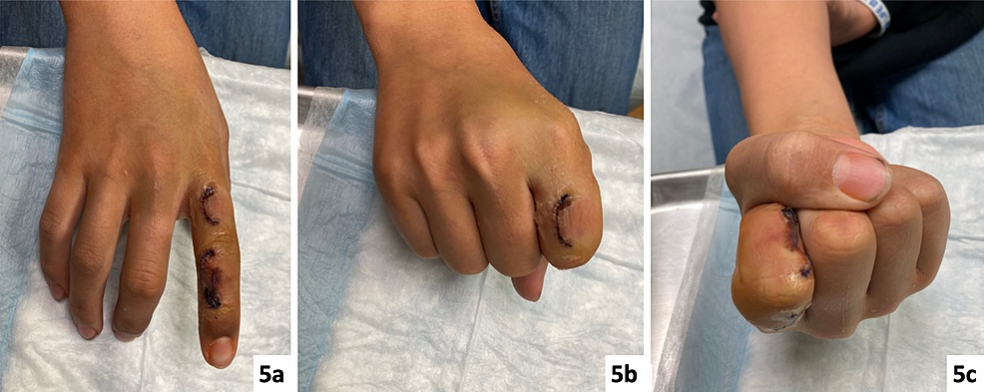
(5a-5c) Postoperative images of the right index finger taken at the two-month follow-up appointment

Based upon these results, the patient was diagnosed with a VM. Following surgery, the patient had an uneventful postoperative course with full range of motion in his index finger at the two-month follow-up (Figure 5).

## Discussion

VMs are present at birth but can be difficult to diagnose at the time until the child has grown up and reached adulthood. These lesions present clinically as blue, soft, compressible lesions that may contain hard calcifications, known as phleboliths, caused by the stagnation of blood in these lesions.

Almost all VMs are sporadic, with one study reporting that 1.2% of VMs were inherited. GVMs, on the other hand, are typically familial, with one series reporting a frequency of inheritance at 63.8% [4]. Until recently, our knowledge of the genetic basis of familial forms has been limited, but with the development of next-generation sequencing, mutations associated with many lesions have been identified. Mutations involved in vascular malformations most commonly involve tyrosine kinase receptor signaling. VMs have previously been found to be caused by eight different genes including TIE2 and PIK3CA, while GVMs are caused by mutations in the glomulin gene [1]. Mutations in the same gene have been found to have varying clinical manifestations, such as that seen with TIE2 mutations. Mutations in TIE2 are capable of causing sporadic VMs, cutaneomucosal autosomal dominant VMs, and blue rubber bleb nevus syndrome - a rare non-hereditary condition associated with multifocal, small (<2 cm) VMs affecting the skin, soft tissue, and gastrointestinal tract [8-11].

VMs and GVMs are histologically and molecularly distinct vascular anomalies, and due to differences in treatment modalities, clinical criteria have been set forth to better distinguish between the lesions to direct management. The majority of VMs can be diagnosed clinically through patient history and physical exam [4]. VMs are typically isolated, bluish mucosal or subcutaneous lesions that shrink under external pressure [1]. The pain associated with VMs is often worse in the morning, and a correlation has been made with the increased pain of VMs during the onset of puberty as well as during menstrual cycles [4]. In contrast, characteristics that suggest the lesion in question is a GVM, especially if it is located on an extremity, include a cobblestone-like appearance, hyperkeratosis, and pain on compression [1].

VMs are known to cause a chronic coagulopathy, and as such, are capable of causing thrombosis or bleeding. Due to this chronic coagulopathy, laboratory studies including D-dimer and fibrin levels can be useful when evaluating for a possible VM as these are often elevated [2]. Previous studies have reported the sensitivity and specificity of D-dimer testing for VMs to be 42.6% and 96.5%, respectively [12]. If necessary, hand-held Doppler examination can be utilized to rule out fast-flow lesions and appreciate enlargement of VMs with dependent positioning. Ultrasound can also help to confirm diagnosis of a VM with characteristic findings of hypoechoic spaces with septations and no flow on color Doppler. Phleboliths may be reported on ultrasound as hyperechoic with acoustic shadowing [13,14]. Other imaging studies such as MRI may demonstrate infiltration of surrounding structures, phleboliths, and an anomalous venous drainage system [13].

The ability to differentiate between GVMs and VMs is paramount in planning successful treatment. While compression garments often improve the pain associated with VMs located in extremities, they can worsen the pain due to a GVM. Surgical excision of GVMs is generally quite successful, while complete excision of a VM is often difficult due to the extensive involvement of tissues and deep structures surrounding the lesion [4]. The efficacy of sclerotherapy has been previously studied for both VMs and GVMs and has been shown to be ineffective in patients with a GVM. Conversely, sclerotherapy has been demonstrated to be much more effective in shrinking VMs [15,16].

This case presented here demonstrates an anomalous presentation based on previously defined clinical criteria of a VM. This adolescent male presented with multiple lesions involving the right, dorsal index finger, as well as another overlying the right olecranon. Further evaluation with ultrasound and radiographic imaging suggested GVMs with slow-flow lesions and no phleboliths noted. Based on these suggested findings of GVMs, the patient was not considered a candidate for sclerotherapy. Surgical excision of these superficial lesions with no surrounding tissue involvement was easily accomplished, further suggestive of GVMs. Upon histopathological examination, however, no glomus cells were seen, and the lesions were determined to be VMs. As such, this case represents an unusual presentation of VMs occurring in a 14-year-old male with notable deviation from criteria previously put forth to distinguish between VMs and GVMs.

## Conclusions

Due to the wide variety of presentations and distinct differences in the management of VMs versus GVMs, clinical diagnosis is essential. VMs tend to be found as solitary lesions, that may involve deeper structures, and are not painful to compression. Phleboliths are often present on radiographic imaging. GVMs tend to occur as multiple lesions involving the superficial skin and subcutis that are painful to compression. Glomus cells are demonstrated on histopathology and there is no evidence of phleboliths on radiographic imaging. This case represents a mixed presentation and deviation from the clinical criteria previously used to differentiate a sporadic VM from a GVM.
